# Pathogen elicitor peptide (pep), systemin, and their receptors in tomato: sequence analysis sheds light on standing disagreements about biotic stress signaling components

**DOI:** 10.1186/s12870-024-05403-y

**Published:** 2024-07-30

**Authors:** Alice Kira Zelman, Yi Ma, Gerald Alan Berkowitz

**Affiliations:** Department of Plant Science and Landscape Architecture, University of Connecticut, Storrs, CT 06269 USA

## Abstract

Peps are endogenous damage-associated polypeptides that evoke defense responses in plants. Like other damage-associated molecular patterns, Pep signals are transduced by receptors. PEPRs are the receptors that transduce Pep danger signals. This paper identifies new putative Peps in the *Solanaceae* (including *Solanum* spp., *Nicotiana* spp., and *Petunia* spp.) and *Coffea* and explores their properties. Using these newly identified Peps we derive sequence logos that present a refinement of the current understanding of the importance of specific residues in the Pep signaling molecules in *Solanaceae*, including several arginines, prolines that restrict peptide’s conformations, and C-terminal asparagine. We examine the degree of disorder in Pep, which is likely important to the mechanism of Pep perception. This work also calls into question some of the evolutionary relationships between Peps in Solanaceae and specific *Arabidopsis* Peps published in previous literature, culminating in a conclusion that SlPep should not be named SlPep6 due to the lack of conservation of protein sequences in AtPROPEP6 and SlPROPEP, and that SlPep probably does not have two receptors in tomato, based on phylogenetic analysis. Our analyses advance understanding of the Pep signaling system in *Solanaceae*.

**Supplementary Information:**

The online version contains supplementary material available at 10.1186/s12870-024-05403-y.

## Introduction

Plant defense requires detection of dangers such as herbivore damage and infection by pathogenic microorganisms. Cellular damage due to herbivores and pathogens causes damage-associated molecular patterns (DAMPs) to activate defense signaling, which initiates active defense against the threat [1]. DAMPs are detected by receptor proteins that transduce the danger signal. While some DAMPs function in the homeostatic state, and only become danger signals during an attack, other DAMPs are specifically induced by biotic stress; this latter type is called inducible DAMPs (iDAMPs) [2]. Systemin and Plant elicitor peptides (Peps) are two types of peptide hormones that function as iDAMPs [3].

Systemin is found in tomato and some of its close relatives; Peps, however, are found in myriad plant species in multiple angiosperm families [3]. Systemin has been shown to initiate defense responses against biotic stresses (reviewed in [4]. While some Peps affect development and abiotic stress responses [5–9], the majority of research has focused on their roles as iDAMP signaling molecules in responses to biotic stresses. In addition to their roles as iDAMPs, both Peps and systemin are post-translationally processed polypeptides. Peps are the presumed mature, signaling C-terminal portions of longer proteins called PROPEPs. Likewise, systemin is a mature C-terminal portion of a longer protein called Prosystemin.

Lori et al. found that distantly related plants have PROPEP sequences, and Pep sequences, with minimal conservation, but identified specific functional residues of presumed PROPEPs by comparison of intrafamily-conserved residues [10]. They aligned several solanaceous Peps previously identified by Huffaker et al. [11] as well as PROPEPs they identified from two additional wild tobacco species. Their analysis led them to rename the solanaceous Peps with the suffix “6” to indicate that the Peps of *Solanaceae* were most closely related to AtPep6 (among the *Arabidopsis* Peps); for example, tomato Pep was renamed SlPep6 [10]. The phylogenetic analysis on which they based this decision bears examining. The bootstrapped tree published by Lori et al. lists the number of bootstraps grouping the solanaceous Peps most closely with AtPROPEP6 as 1000 of 1000. Given the reported lack of conservation of PROPEPs in different families, this is an improbable result and bears reproduction for confirmation. We reveal a phylogenetic analysis that did not substantiate Lori et al.’s analysis, from which we conclude that SlPep should not be renamed SlPep6.

Sequence logos can graphically depict conserved areas of sequences [12]. They are derived from multiple sequence alignments (MSAs) of related sequences. Lori et al. [10] used four non-identical Pep sequences (one *Nicotiana* and three *Solanum*) to determine a solanaceous Pep consensus sequence logo. We and Huffaker et al. [11] have identified additional solanaceous Peps (see Methods). Generating a consensus from all of these Peps gives additional power to generalize what parts of the sequences are conserved among the whole family. Sequence logos that may not only display sequence conservation but also adjust estimates of conservation based on the likelihood of a particular amino acid occurring at each position in an MSA are more powerful than logos that only average positions in an MSA [13]. We assess the conservation of solanaceous Pep residues using this technique to further knowledge about the importance of specific residues in these peptides. We also present an exploration of the similarities between systemin and Pep to further knowledge of these important signaling molecules.

Gaining a comprehensive understanding of Pep signaling in solanaceous crops necessitates the identification of SlPEPR(s). Lori et al. [10], Xu et al. [14], and Rahman et al. [15] identified AtPEPR1/AtPEPR2 homolog SlPEPR. Rahman et al. concluded that a second protein, SlGC17, was also a PEPR. Since there are questions about the identity of the Pep receptor(s) in tomato and how Pep in tomato is related to Peps in other species, our research contributes a more rigorous study of the phylogeny and identities of PROPEPs in solanaceous species and related plants, and of PEPR and its homologs in tomato.

## Results

### SlPep is a disordered peptide with conserved residues

Coffee and nightshades reside in the asterid clade of *Pentapetalae*, and therefore share a more recent common ancestor with each other than with *Arabidopsis*, which is a rosid [16]. The clade-oriented database solgenomics.net [17] has proteomic and/or genomic databases for cultivated and wild solanaceous plants including *Solanum*, tobacco (*Nicotiana*), pepper (*Capsicum*), and *Petunia* species. Sol Genomics also has resources for coffee, a non-solanaceous plant [18]. We identified previously unknown putative Peps in solanaceous species and *Coffea* species. We refer to the Peps we studied as [Species identifier][Pep], e.g. SlPep, unless otherwise specified, because S. *lycopersicum* and other solanaceous plants have so far only one Pep identified in each species [10, 11]. We identified an exception, in *S. chilense*, which has 2 predicted Peps.

**Fig. 1:**
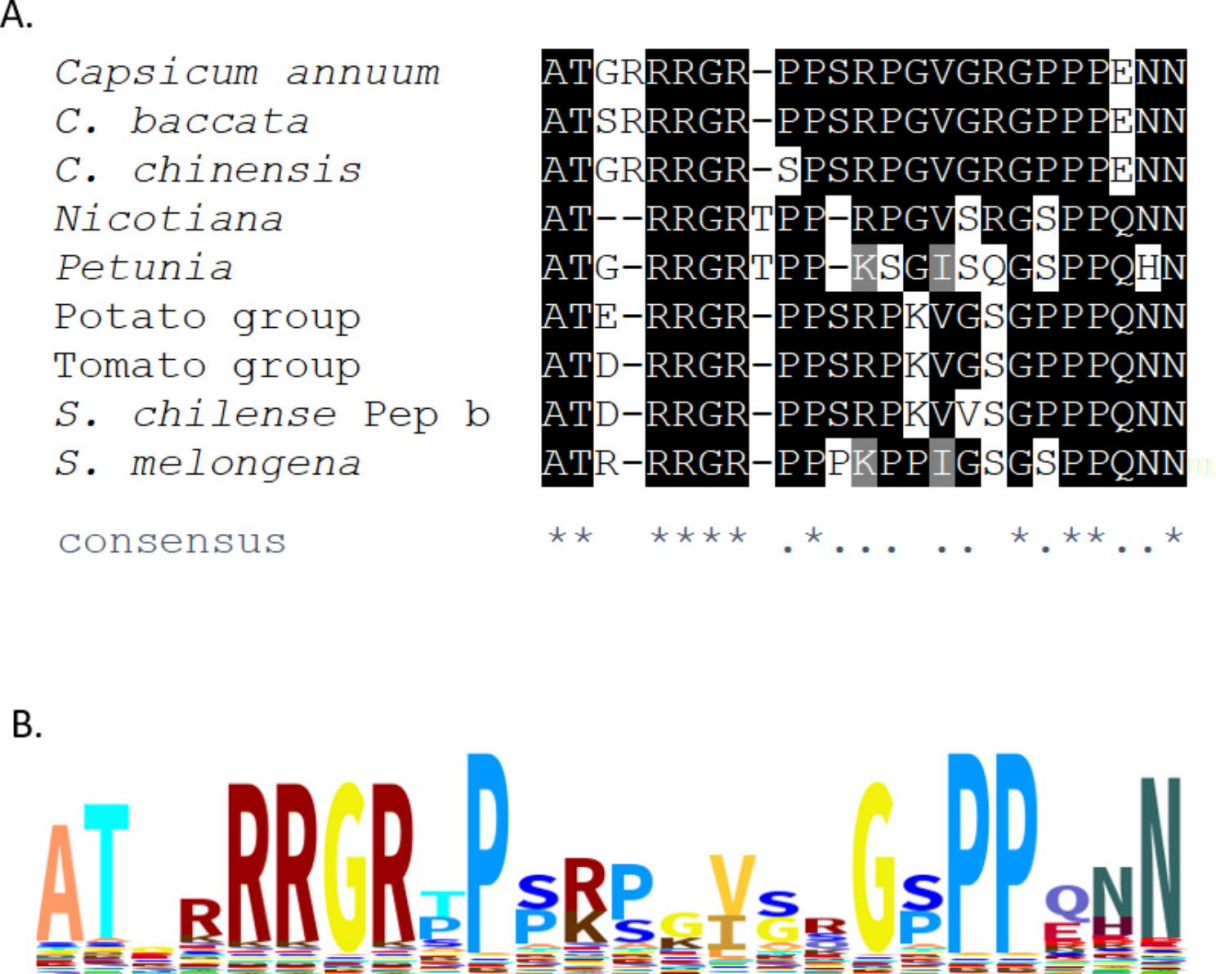
Solanaceous Peps show sequence conservation. A. MSA of Peps identified in Solanaceae. Nicotiana sequence is that of the identical Peps in N. tomentosiformis, N. sylvestris, N. benthamiana, N. tabacum, and N. attenuata. N. otophora’s putative Pep is not included. Petunia sequence is that of the identical Peps in P. axillaris and P. inflata. Potato group is Solanum commensoni and S. tuberosum. Tomato group is S. lycopersicum, S. pimpinellifolium, S. chilense, and S. pennelli. Residues which are identical in at least 8 of the 9 sequences are highlighted in black. Residues which are identical in 7 of the 9 sequences are highlighted in gray. The consensus line at the bottom shows “*” for each position in the alignment that is identical in every sequence, and “.” for each position that is identical in 7 or more of the sequences. S. chilense Pep b differs from the other tomato Pep sequences at the position indicated with a “╪” symbol., where it has an V instead of a G residue. B. Pairwise alignment (see Methods and Materials) of the two putative PROPEPs identified in Solanum chilense. Matches are highlighted; mismatches and gaps in black text on white.

First we made an MSA of solanaceous Peps, including predicted and experimentally validated sequences (Fig. 1A). Overall, we found a very high degree of conservation in the solanaceous Pep orthologs. Of 25 total positions in the MSA, 11 positions had identical residues in at least 8 out of the 9 included sequences, and 20 positions had identical residues in 7 out of the 9 sequences. This percentage of conservation is high in comparison to the AtPeps, and an MSA of AtPep1-8 and the solanaceous Peps shows a much lower degree of conservation (Supplementary Fig. 1). In this MSA of *Arabidopsis* and solanaceous Peps only a small percentage of positions have identical or similar residues. Of particular interest is the alignment of SlPep with the AtPeps, especially AtPep6, since it has been claimed that AtPep6 is the closest ortholog to SlPep [10], a contention which will be discussed later.

We generated a profile Hidden Markov Model (HMM) from the MSA of solanaceous Peps (MSA depicted in Fig. 1A). Subsequently we created a sequence logo from this profile HMM (Fig. 1B). According to this model, proline residues contribute most to the sequence conservation in this model, which is reasonable when one considers that prolines rigidly restrict the backbone of a polypeptide change, drastically constraining the shape of the polypeptide. Proline-rich regions change little in conformation when they bind to other proteins; they have a concomitantly lower decrease in conformational entropy compared with other interactions, and therefore favorable binding properties. Proline-rich regions are important to protein-protein interactions [19]. Systemin is notably proline-rich [20]. Glycine residues have the most freedom of movement, and there are several conserved glycine residues in the solanaceous Peps. Arginines and the final asparagine also are clearly conserved among the solanaceous Peps, raising the possibility that polar and/or ionic receptor-ligand interactions are a conserved feature of the Pep-PEPR system in *Solanaceae*. In fact, among the 75 predicted or known Peps listed by Lori et al. [10], all but one sequence have two or more charged or polar residues among the final three (N-terminal) residues. The last residue of AtPep1 was shown to be important for Pep-PEPR binding, and mutation of this residue drastically compromised this binding [21].

**Fig. 2:**
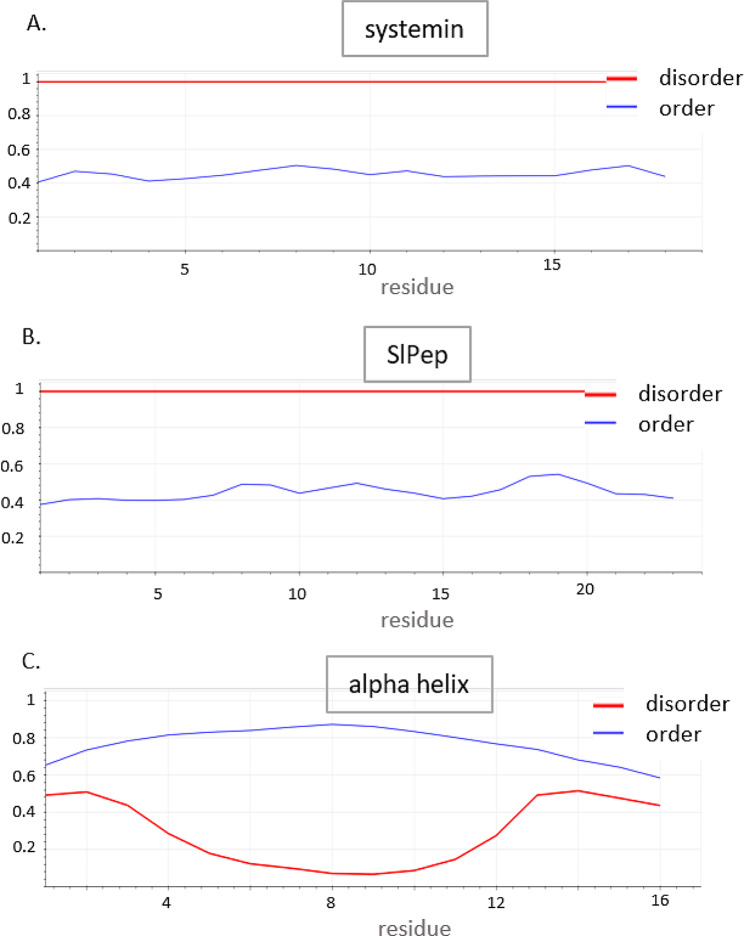
The disordered nature of SlPep and systemin compared with an alpha helical peptide. (A) Prediction of systemin’s order/disorder as visualized by Metapredict (Emmenecker, Griffith, and Holehouse, 2021). (B) SlPep order/disorder prediction. The slight increases in order prediction correspond to the proline residues in the sequence. (C) Order/disorder prediction of a 16-residue polypeptide designed to have optimal alpha helical secondary structure by Petukhov et al. (2009). The sequence of the alpha helical polypeptide is LELLLRLLLLLLLGGY

**Fig. 3:**
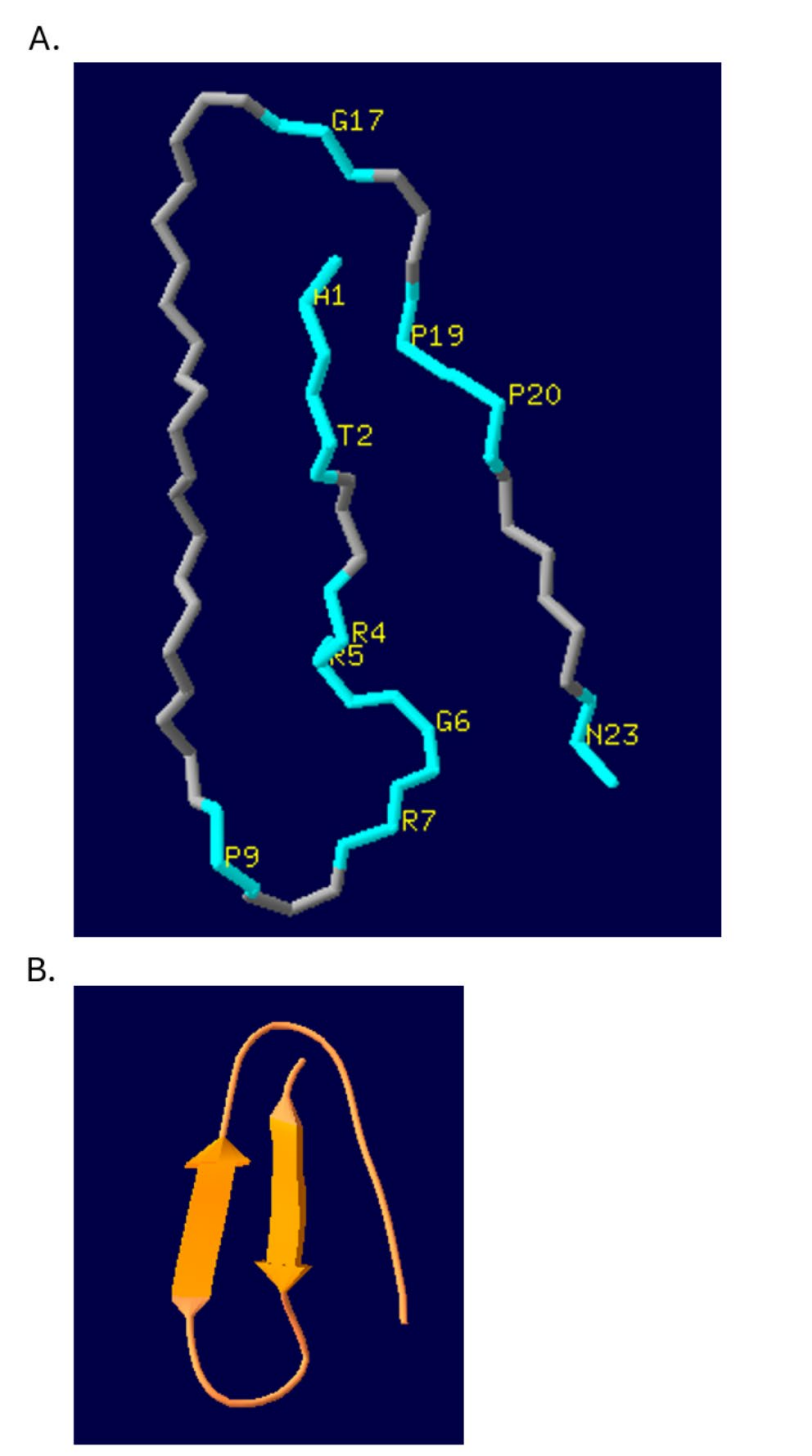
Best fit 3D conformational model of SlPep in solution. A. Conformation of SlPep with conserved residues colored blue. The conserved residues are labeled in yellow, and are marked by * in the consensus line in Fig. 1A. The N-terminus and C-terminus are located at A1 and N23 respectively. There are many probable conformations for a dynamically disordered polypeptide in solution so this depiction is best thought of as a snapshot.B. Ribbon diagram of SlPep in solution. Arrows indicate transient secondary structure

Chemical, biophysical, and computational evidence shows that Prosystemin is an intrinsically disordered protein, and the 18 residue C-terminal portion that comprises the mature signaling peptide is also disordered; the disordered nature of systemin is important to its activity [22]. Systemin shows consistent disorder across its length (Fig. 2A). Metapredict indicates that SlPep is also disordered (Fig. 2B), with order/disorder predictions nearly the same along the lengths of the sequences of SlPep and systemin. SlPep shows slight rises in order prediction values corresponding to its proline residues. For comparison, Fig. 2C shows the order/disorder predictions by Metapredict for an alpha-helical 16-residue polypeptide; the prediction of disordered state is low across the entire sequence. PEP-FOLD3 [23] simulation showed that there are many possible structures of SlPep, as is expected of a disordered protein; the best model is shown in Fig. 3. In solution this polypeptide is predicted to be a dynamically disordered molecule; disordered regions are advantageous in binding interactions [24]. AtPep1 was shown to be in an extended structure when bound to AtPEPR1 [21], suggesting that SlPep may be extended when bound to SlPEPR.

Within genera, Pep sequences are quite conserved. *P. axillaris* and *P. inflata* are species from the two different major clades of *Petunia* [25]. Their predicted Pep sequences were found to be identical. Of the *Nicotiana* species, all had identical predicted Peps except for *N. otophora.* All three predicted *Capsicum* Peps were different from each other, but differed at only two positions in their polypeptide sequences. There was more diversity among the sequences in *Solanum.* The wild potato *S. commersonii* and its cultivated relative *S. tuberosum* have the same predicted Pep sequence. *S. commersonii’s* PROPEP is predicted from a *de novo* genome assembly project (NCBI BioProject Accession: PRJNA655804).

**Fig. 4:**
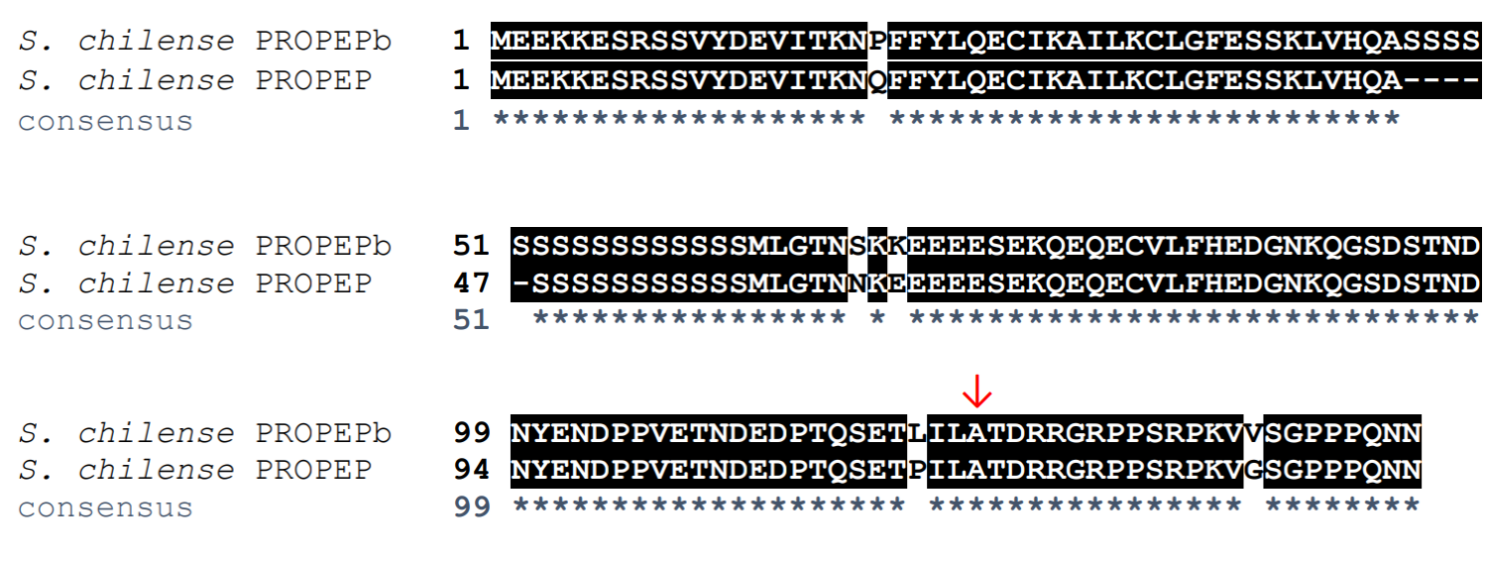
Pairwise alignment of the two putative PROPEPs identified in *Solanum chilense*. Matches are highlighted; mismatches and gaps in black text on white. The consensus line at the bottom shows “*” for each position in the alignment that is identical. “-” indicates a gap at that position in the alignment for that row’s sequence. Arrow (↓) indicates the predicted beginning of the mature Pep polypeptide sequence

Interestingly, *S. chilense* has two predicted PROPEP sequences that would yield non-identical Pep sequences (SOLCI004017000 and SOLCI003173400). Figure 4 shows a pairwise alignment of these two PROPEPs, whose protein sequences are 97% identical. *S. chilense* and *S. lycopersicum* have the same ploidy (level of genome duplication) and chromosome number (2n = 24), suggesting that the two PROPEP sequences reported in *S. chilense* are the result of either gene duplication or heterozygosity, not a whole genome duplication. One *S. chilense* Pep (SOLCI004017000) has an identical sequence to the tomato group’s Pep (that of *S. lycopersicum*,* S pimpinellifolium*, and *S. pennelli)*, and one (SOLCI003173400) differs by one amino acid (ScPROPEPb has a V where ScPROPEP has a G, as seen in Fig. 4 – the one residue that is not marked with a “*” after the beginning of the Pep sequences indicated by an arrow above the MSA. ). We tentatively call this latter sequence *S. chilense* Pep b (sequence beginning at the arrow at the C-terminal end of the top sequence in Fig. 4). It remains to be determined whether the two sequences represent two haplotypes of the same gene locus, or two paralogous genes; the publication describing the draft genome notes that the individual plant sequenced was heterozygous due to the compulsory outcrossing in this species, and the high level of fragmenting in the genome prevented comparison of chromosomal rearrangements with *S. lycopersicum* and *S. pennelli* [26].

**Fig. 5:**
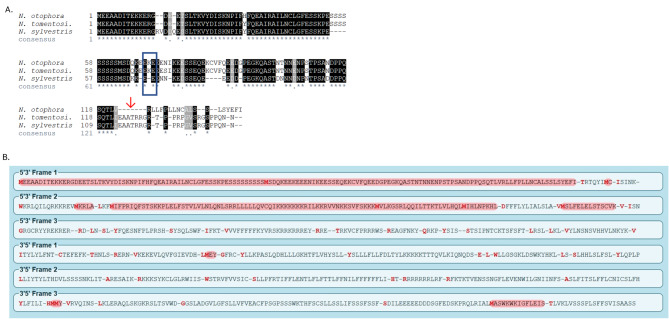
**A**. MSA of predicted PROPEPS from *Nicotiana otophora*,* N. tomentosiformis*, and *N. sylvestris*. The alignment of putative PROPEPS (see Methods and Materials) shows highly similar protein sequences overall, but highly dissimilar predicted Pep sequences. Identical (matching) residues are highlighted in black, and residues which are chemically similar are highlighted in gray. The consensus line at the bottom shows “*” for each position in the alignment that is identical and “.” for each position that has residues with similar chemical properties. “-” indicates a gap at that position in the alignment for that row’s sequence. Arrow (↓) indicates the predicted beginning of the mature Pep polypeptide sequence; note the lack of high-quality alignment after this point. Blue box shows “EKE” motif noted in Huffaker et al., 2011. **B**. Possible open reading frame (ORF) translations for putative PROPEP homolog in *Nicotiana otophora*. The top reading frame (5’3’ Frame 1) produces a single protein sequence that aligns well with *N. tomentosiformis* PROPEP (top sequence in Fig. 4B). 5’3’ Frame 2 produces a protein sequence that has negligible similarity to PROPEP and could therefore not produce a meaningful alignment(data not shown). The other four possible ORF translations have negligible similarity to the PROPEPS in *Nicotiana* spp. along their entire length; they do not produce proteins of sufficient length to make comparisons

Several species’ predicted Peps were not conserved. *N. otophora*’s closest wild tobacco relative is *N. tomentosiformis* [27]. The putative PROPEP sequences from these two species share a high degree of similarity and are clearly homologs: the PROPEP sequences excluding the C-terminal putative functional domain are 97% identical, with no gaps predicted in an alignment of *N. otophora* and *N. tomentosiformis* PROPEPs (the top two sequences in the MSA in Fig. 5A). A related species, *N. sylvestris*, has a predicted PROPEP that aligns well with the *N. otophora* and *N. tomentosiformis* sequences with some gaps (the bottom sequence in Fig. 5A). However, nearly at the precise point at which the presumed functional domain starts – that is, the beginning of the Pep sequence (shown by an arrow in Fig. 4B) – there is a preponderance of gaps in the MSA of PROPEPs of *N. otophora*,* N. tomentosiformis*, and *N. sylvestris*. This indicates that this region of the *Nicotiana* PROPEPs is essentially too divergent to align. The PROPEPs in *N. tomentosiformis* and *N. otophora* contain an “EKE” motif. This motif was found in both *Arabidopsis* AtPROPEP1 and maize ZmPROPEP1 [28]. (The motif is boxed in Fig. 5A.) In the *N. otophora* genomic PROPEP sequence, there is no potential reading frame from which the putative protein sequence is derived that could produce a better pairwise alignment, indicating that a frame shift was not responsible for this lack of similarity in Pep sequences (Fig. 5B). Given this, we hypothesize that *N. otophora* Pep is dysfunctional, but since only one genome is available, this is uncertain.

**Fig. 6:**
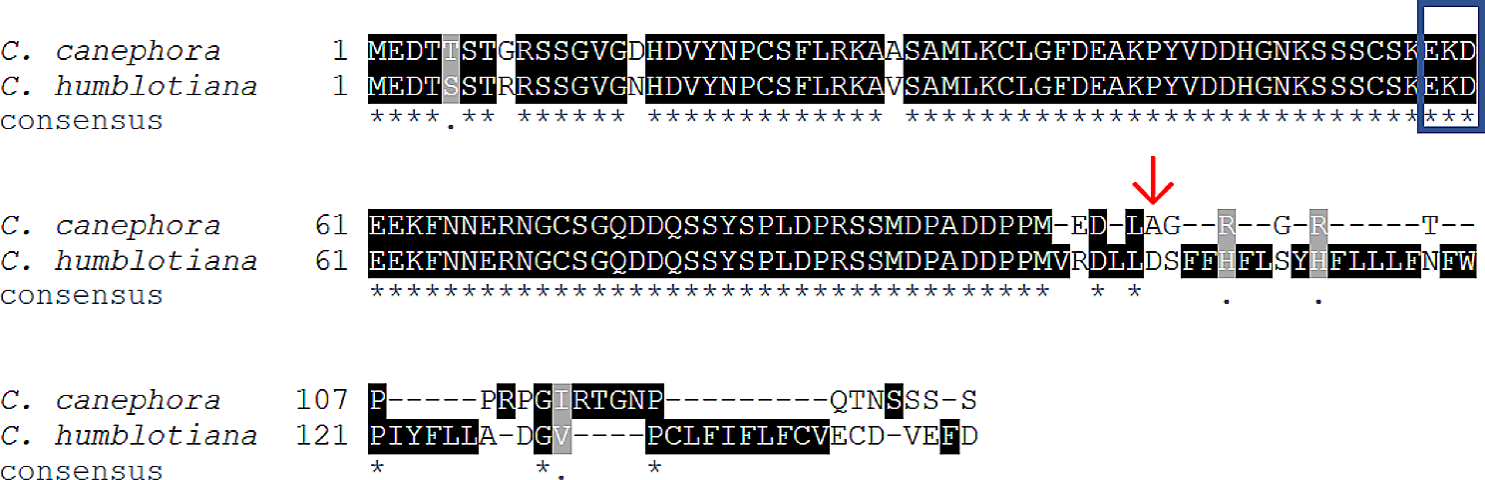
Predicted PROPEPS from *Coffea canephora* and *C*. *humblotiana*. The alignment of putative PROPEPS (see Materials and Methods) shows highly similar protein sequences overall, but highly dissimilar predicted Pep sequences. Identical (matching) residues are highlighted in black, and residues which are chemically similar are highlighted in gray. The consensus line at the bottom shows “*” for each position in the alignment that is identical and “.” for each position that has residues with similar chemical properties. “-” indicates a gap at that position in the alignment for that row’s sequence. The “EKE” motif (Huffaker et al., 2011) is not present but a similar “EKD” motif is boxed in blue. Arrow (↓) indicates the predicted beginning of the mature Pep polypeptide sequence; note the lack of high-quality alignment after this point

**Fig. 7:**
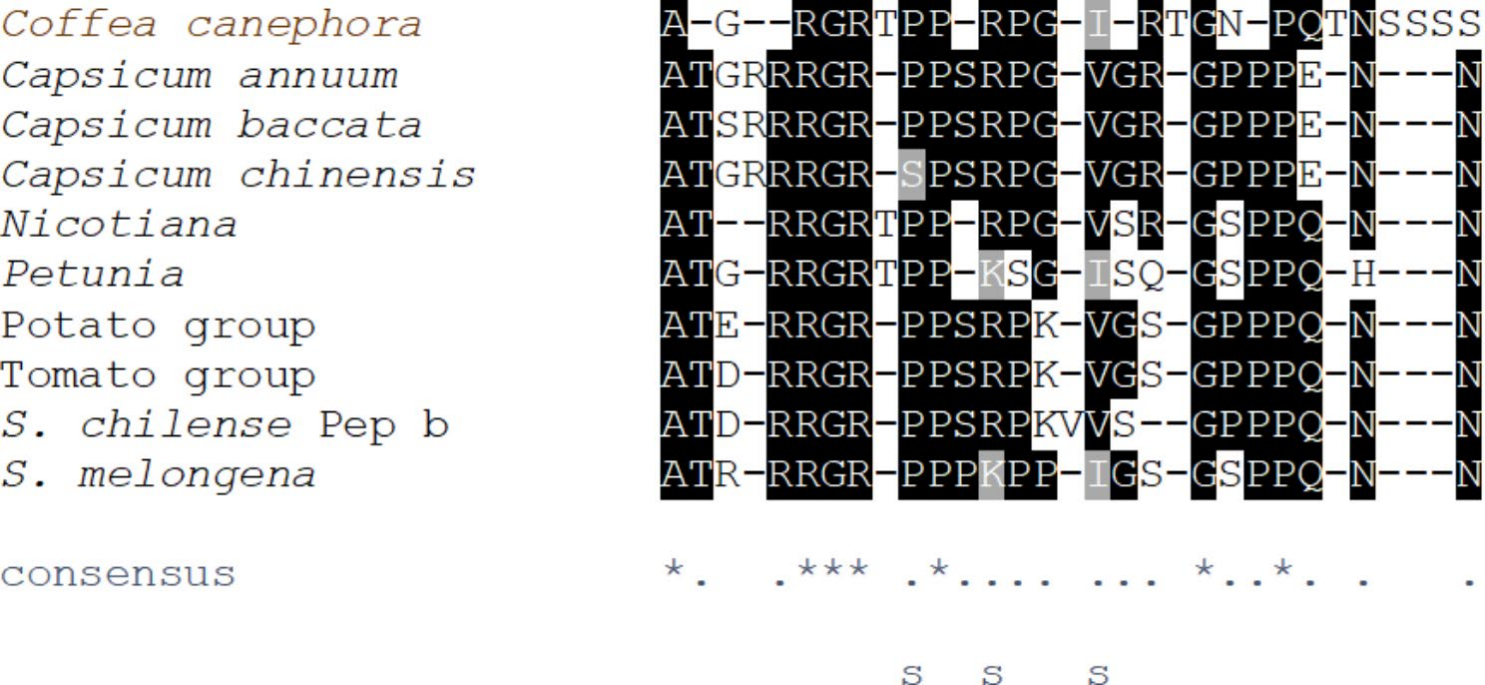
Alignment of the putative Peps in *Coffea canephora* with Peps of *Solanaceae*. (See Materials and Methods). Residues which are identical in at least 7 of the 10 sequences are highlighted in black. Residues which are chemically similar to the most frequent amino acid at that position are highlighted in gray, namely K which is similar to R, and I which is similar to V. Sites at which the *Coffea canephora* residue was identical to the most frequent amino acid in the *Solanaceae*, or chemically similar to it, are marked with an “s” below the consensus line. *Nicotiana* sequence is that of the identical Peps in *N. tomentosiformis*, *N. sylvestris*, *N. benthamiana*, *N. tabacum*, *and N. attenuata*. *N*. *otophora’s* putative Pep is not included. *Petunia* sequence is that of the identical Peps in *P*. *axillaris* and *P*. *inflata*

Cultivated and wild coffees had a similar interesting disparity in the putative Pep sequences we found. Using the *C. canephora* PROPEP sequence we identified earlier, we queried the draft genome of *Coffea humblotiana* for a putative PROPEP. This yielded a sequence that is 93% identical in the N-terminal portion of the protein (before the putative Pep sequence). The coffee PROPEPs do not have the strict “EKE” motif reported for other species, including in *Solanaceae*, by Huffaker et al. [11], but did have the chemically similar “EKD” motif (shown by the blue box in Fig. 6). *C. canephora*’s putative Pep surprisingly aligns better with the solanaceous Peps than with the predicted *C. humblotiana* Pep sequence; it shares several conserved sites (Fig. 7). Of the 11 positions that were identical among all solanaceous sequences, six were also identical in *C. canephora* (marked with “*” in the consensus line under the alignment in Fig. 7). At an additional three positions, the *C. canephora* Pep sequence was identical to a conserved residue that was present in less than 100% but more than 90% of solanaceous Peps studied.

**Fig. 8:**
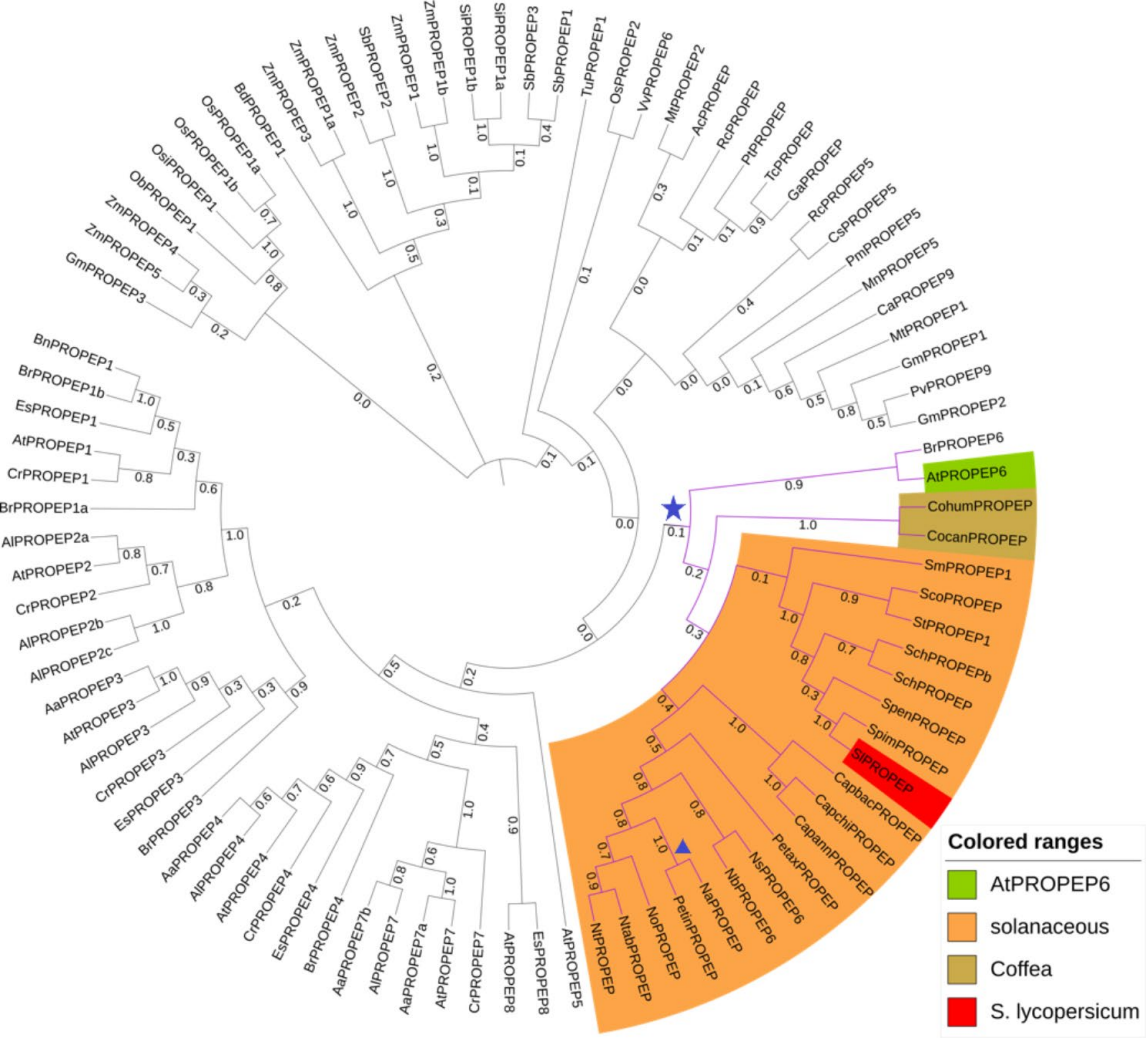
Relationship between PROPEPs in plant species, derived from analysis of protein sequences. The evolutionary history was inferred by using the Maximum Likelihood method and JTT matrix-based model. The tree with the highest log likelihood (-19113.42) is shown. The proportion of trees in which the associated taxa clustered together is shown next to the branches. Initial tree(s) for the heuristic search were obtained automatically by applying Neighbor-Join and BioNJ algorithms to a matrix of pairwise distances estimated using the JTT model, and then selecting the topology with superior log likelihood value. This analysis involved 91 amino acid sequences. There were a total of 312 positions in the final dataset. for the star symbol marks the clustering of AtPROPEP6 and the solanaceous PROPEPs. The triangle symbol marks the branch with PetinPROPEP and NaPROPEP

Previously AtPep6 was reported to be the closest ortholog to SlPep. The published phylogram reports that 1000 of 1000 bootstraps clustered AtPROPEP6 and SlPep together (and that the closest Arabidopsis ortholog to SlPROPEP is thus definitively AtPROPEP6) [10]. We found insufficient bootstrap support for this hypothesis: in our analysis only approximately 10% of bootstraps clustered AtPROPEP6 with the PROPEPs from the analyzed asterids, namely *Solanaceae* and *Coffea* (Fig. 8). This does not disprove the hypothesis that AtPROPEP6 is the AtPROPEP most closely related to SlPep and the other solanaceous Peps, but it certainly casts doubt on it. Supplementary Fig. 1 shows an MSA of the solanaceous and *Arabidopsis* Peps. In a pairwise comparison of AtPep6 and SlPep, 9 residues are identical or have amino acids with similar properties. However, other AtPeps have almost as much similarity to SlPep, especially AtPep1 and AtPep2. The similarity of the short Pep sequence cannot be considered significant in considering conservation; rather, the full-length PROPEPs must be considered in an analysis of conservation.

The solanaceous PROPEPs appear most closely related to each other, although here too there is low bootstrap support for the presented tree – while it is the best fit in our analysis, only approximately 30% of 1000 bootstraps clustered all 19 solanaceous sequences on a single monophyletic branch (Fig. 8). Given that most solanaceous plants have only one identified PROPEP, it is very likely that all of these solanaceous PROPEPs are derived from a single ancestral PROPEP sequence in their common ancestor, and the low bootstrap support reflects the relatively low sequence conservation in PROPEPs in general. Within solanaceous genera, the bootstrap support for clustering PROPEPs was often stronger. For example, *Capsicum* sequences appeared on a single branch in nearly all iterations. SlPROPEP was almost always located on a branch with 6 of 7 *Solanum* PROPEPs, the exception being *S. melongena*. Unexpectedly, and probably spuriously, there was strong support for *Petunia* sequences appearing on separate branches among the *Nicotiana* sequences – *P. inflata* and *N. attenuata* clustered in nearly all iterations. As expected, AtPROPEP6 did cluster most frequently with BrPROPEP6, another brassicaceous PROPEP6 (approximately 90% of generated trees). The full phylogenetic tree of the PROPEPs we analyzed is published at, and may be interactively explored at, https://itol.embl.de/shared/CjjCRJ1zZHdL hosted by the Interactive Tree of Life [29].

### There is likely only one SlPEPR in *S. Lycopersicum*

Pep signaling is a conserved pathway in the plant kingdom [30], so a high degree of conservation of PEPRs is expected across plant families as well. Here we explore the phylogenetic relationships between PEPRs and related sequences. Tomato is a model organism, but scientific knowledge of tomato genes and their associated transcripts and proteins is less complete than that of the “gold standard” of plant biology, *Arabidopsis*.

We performed an analysis to flesh out the evolutionary relatedness and history of SlPEPR and related genes. The “first pass” Xu et al. [14], Lori et al. [10], and Rahman et al. [31] performed in their initial identification of putative SlPEPRs was to search using the sequence(s) of known gene(s) of interest as a query against a database of sequences in the species of interest (usually a variant of BLAST [32]), which retrieves similar sequences in other species.

There is a disagreement about how many PEPRs exist in tomato: Xu et al. and Lori et al. stated that the gene identified in Sol Genomics as Solyc03g123860 was the SlPEPR, and no further PEPRs were identified, while Rahman et al. put forward two candidate SlPEPRs, Solyc03g123860 and another gene identified in Sol Genomics as Solyc03g112580. We used the comprehensive *Arabidopsis* resource TAIR [33] to BLAST using these tomato sequences as queries. The best hit for SlPEPR is the AtPEPRs, but the *Arabidopsis* best-hits for SlGC17 in our pBLASTx search were ROOT MERISTEM GROWTH FACTOR (RGF) receptors whether cDNA or amino acid sequences for SlGC17 were used as query templates. All five members of this clade had e-values of 0, which indicates certainty that they are homologous. RGF1-INSENSITIVE 4 (AtRGI4) (TAIR ID: AT5G56040) and RGF1 INSENSITIVE 3 (AtRGI3) (TAIR ID: AT4G26540) had the highest (and nearly identical) bit scores of 1031 and 1012 respectively. All five RGI proteins are LRR-RLKs and are receptors for the signaling peptide ROOT MERISTEM GROWTH FACTOR 1 (AtRGF1), a key regulator of root meristem activity [34]. RGI4 is also a receptor for ROOT MERISTEM GROWTH FACTOR 7 (RGF7), which triggers innate immunity [35]. Importantly, when the AtRGI4 cDNA and amino acid sequences were used as a template for the BLAST variants blastn (for nucleotide sequences) and blastp (for protein sequences), the best hit was not SlGC17 but an LRR-RLK on chromosome 7 (Solyc07g065860). The latter protein does not appear in any publications, to the best of our knowledge. This ambiguity is not surprising, given the large number of members and similar ligand-binding activities and cellular activities of the LRR-RLKs. SlGC17 was automatically annotated with the Gene Ontology (GO) terms GO:0001653 – peptide receptor activity and GO:0004674 – protein serine/threonine kinase activity. AtRGI4 is annotated with the same GO terms. Solyc03g123860.2 (SlPORK1) is annotated with the GO term for peptide receptor activity but not protein serine/threonine kinase activity. However, it is tagged “Computational annotation: Receptor-like kinase (AHRD V1 **** A7VM19_MARPO); contains Interpro domain(s) IPR002290 Serine/threonine protein kinase.” As a step in confirming this putative PEPR, its protein domains were classified. This putative tomato PEPR is predicted to comprise a region containing multiple LRRs, a transmembrane domain, and intracellular kinase and GC domains: the same arrangement of domains as the AtPEPRs have, lending credence to its identification as a Pep receptor. However, there are many other receptor kinases and receptor-like kinases in plants that share these features, so mere similarity cannot prove that the putative SlPEPR is a bona fide Pep receptor. We therefore constructed a phylogram from selected LRR-RLKs (Supplementary Fig. 2). Rahman et al. did not specify the parameters that they used to generate their MSA(s), but they did indicate the use of ClustalW as the method. In an exploratory series of parameter alterations used by the ClustalW algorithm to try to replicate the tree Rahman et al. published, when including the full complement of LRR-RLK and GC sequences in Supplementary File 3, we were unable to construct an MSA that, when used to construct a phylogram, resulted in the placement of SlGC17 and SlPEPR as the most closely related sequences to each other and to the AtPEPRs on one branch of the tree (data not shown).

Branch placement among AtPEPR1, AtPEPR2, SlPEPR, SlGC17, SlSYR1, and SlSYR2 was identical whether the MSA used was generated by ClustalW or MUSCLE as described in the Methods section. Based on this analysis, we conclude that SYR1 and SYR2 are likely the closest evolutionary neighbors to SlPEPR in the LRR-RLK family in tomato, in agreement with Xu et al. [14].

## Discussion

*Arabidopsis*, the nightshades, and coffee all belong to the class Pentapetalae, which includes about 70% of flowering plant species [16]. This class diverged chiefly into two clades: asterids (including coffee and nightshades) and rosids (including Arabidopsis). Due to the relatively recent divergence of the asterids from other clades of Pentapetalae, homologs in coffee and the nightshades can be expected to have coding sequences more similar to each other than *Arabidopsis* genes are to either. Profile HMMs can be useful in detecting dissimilar homologs. Lori et al. (2015) used HMMER [36]to construct HMMs to find new PROPEPs. Using HMMs to detect homologs recently led to the discovery that the LRR-RLK gene family is larger than previously thought, with many members eluding identification in the past due to gain or loss of domains and structural variation [37]. In the future this approach may help identify orthologs of PROPEPs in plants species, and perhaps determine whether PROPEPs evolved before the advent of angiosperms. Our use of Skylign’s HMM with the additional Peps we identified enabled us to construct a weblogo that highlighted the residues most likely to be important to Pep function. We also found that SlPep has a high degree of disorder, comparable to systemin. Disordered regions typically evolve faster than structured regions, because there is no requirement for consistent packing interactions, but the property of being disordered is conserved among functional regions of e.g. signaling peptides (van der Lee et al., 2014). Therefore, we expect Peps will consistently show constrained disordered properties: conservation of particular residues, and a disordered structure when not bound to a receptor.

Multiple sequence alignments (MSAs) can offer clues about conserved residues in polypeptides. It should be noted that the sequences identified are putative, and the mature Pep signaling peptides might be longer or shorter than these predictions, which are based on alignment with existing Peps. For example, it is unclear if the native Peps in *Capsicum* have an additional alanine residue at their N-terminus after the assumed cleavage from their PROPEP sequences. We used MSAs to show the high overall conservation of PROPEP sequences but lack of similarity of Pep sequences in *Nicotiana otophora* and its closest relatives with identified Peps, *N. tomentosiformis* and *N. sylvestris*, and the same situation in *Coffea canephora* and *C. humblotiana*. Either there are errors in their draft genome assemblies, or *N. otophora* and *Coffea humblotiana* have PROPEPs that yield Peps with no similarity to Peps from their close relatives. When a draft genome is assembled and its genes are predicted, there are often errors in gene structure prediction [38], so the *N. otophora* and *C. humblotiana PROPEP* transcripts should be sequenced to confirm the predicted protein sequences. The activity of the predicted Peps should also be confirmed experimentally to verify their identification as signaling peptides.

It is important to note that the top BLAST hit is not necessarily the most closely related ortholog in the target organism. BLAST works well to identify closely related sequences between comprehensively sequenced genomes but can fail to account for paralogs generated by gene duplication [39]. The “top hit” may be just one paralog of several that must all be considered equally related neighbors to the original query sequence. It is critical to BLAST the best hit back to the original species’ genome before concluding that a sequence is the likeliest ortholog (i.e., confirm it is the “reciprocal best hit” [RBH]). This may be one reason there is currently a disagreement in the literature about the number of Pep receptors in tomato.

Sequence selection for the construction of MSAs is a crucial step in phylogenetic analysis, and the most intellectually challenging, according to Kumar et al. [40]. When sequences contain large duplications, rearrangements, or deletions, the MSA generated may not reflect true evolutionary relatedness. Given the same set of sequences, the MSA method and its parameters are key choices that affect the outcome of the alignment and the subsequent phylogenetic analyses performed using that alignment.

The root of the disagreement about how many PEPRs exist in tomato may lie in the different selection of analyzed sequences by different research groups, as well as the previously discussed methods for generating MSAs used to construct phylogenetic trees. Xu et al.’s analysis used the Neighbor-Joining (NJ) method to construct their proposed phylogeny [14]. NJ is considered a good choice to construct a tree quickly, but other methods have the increased rigor of more time/resource-intensive approaches. Rahman et al. used the Maximum Likelihood (ML) method, which is considered more rigorous and accurate than NJ. Initially, this led us to suspect that Xu et al.’s tree was less likely to reflect true relationships between the sequences. The confidence of the tree produced by Rahman et al. was lower for SlGC17’s placement in the same clade as AtPEPR1, AtPEPR2, and SlGC18/PORK1 [15]. Rahman et al. did not include SYSTEMIN RECEPTOR1 and SYSTEMIN RECEPTOR2 (SlSYR1 and SlSYR2) in their analysis, since these two proteins lack the GC catalytic center motif that they specified, more specifically they lacked a plant GC-specific sequence motif that fits the regular expression [KS] [YF] [GCS] [VIL] [VILFG] [DVIL] [VILADG] [EPVIL] [DVIL] [TVIL] [WST] [PDRG] [KEG] [KR] x{2,3} [DHSE] [31]. Using only proteins that contained a GC motif was reasonable to begin an analysis of GC activity. However, this meant that the authors did not include all potential orthologous genes in their analysis. (It is unclear whether SYR1/2 have GC activity. They are conserved at the position of the reported GC center with respect to SlPEPR and AtPEPR1/2 (Supplementary Fig. 3) but are not captured by the regular expression published by Rahman et al., and therefore a more relaxed regular expression would have identified them. Experimental confirmation or refutation of GC activity would be necessary to be certain.) For this reason, it was important to investigate the LRR-RLKs and GCs that both these papers included in their analysis [14, 15]. Our analysis in this paper does not prove that SlGC17 is not a PEPR, but it does indicate that the attribution of PEPR function to SlGC17 with no experimental evidence is unwarranted. Further studies should delve into the functional analysis and peptide binding activity of SlPEPR and SlGC17 to determine their roles as Pep receptors. Likewise we did not find sufficient support for the previously published assertion that SlPep is most closely related to AtPep6. Notably, most work on Pep signaling in solanaceous species is specifically in tomato. Additional experimental work is needed to advance our knowledge of the Pep-PEPR system in other non-model solanaceous species such as pepper, eggplant, and potato, and myriad crops in other families with uninvestigated Peps.

## Methods

### Sequence identification and selection

Sequences to determine the likely relationship to AtPEPR1 and AtPEPR2 were selected to include those found in the analyses of Xu et al. (2018) and Rahman et al. (2020) and are named accordingly. Tomato sequences specified in Xu et al. [14] and Rahman et al. [15] were downloaded from the Sol Genomics genome database (https://solgenomics.net) [18]. To identify the PEPR gene(s) in tomato, we first used the full-length AtPEPR1 and AtPEPR2 mRNA sequences as a query to identify the likeliest orthologous protein(s) in tomato. We queried SolGenomics [18] full genomes, gene models, and RNA sequences from numerous varieties of tomato and other solanaceous plants. Phytozome [41] was also used for performing *Arabidopsis* to tomato BLAST searches, to repeat the protocol reported by Rahman et al. [31]. Solanaceous putative PROPEP sequences were also downloaded from Sol Genomics as noted in the text. Other sequences were downloaded from NCBI, as noted in text. Using SlPROPEP as a query and a very relaxed E-value cutoff, we identified candidate PROPEPs in all these species for which Pep sequences have not yet been published; Reference genomes for *S. lycopersicum*,* S. pimpinellifolium*,* S. tuberosum*,* S. chilense*,* S. melongena*,* Nicotiana benthamiana*,* N. tabacum*,* N. sylvestris*,* N. tomentosiformis*,* N. attenuata*,* Capsicum annuum*,* C. chinensis*,* Petunia axillaris*, and *P. inflata* were accessed on Solgenomics.net [18]. Because each genome was queried separately, no particular e-value or bit score was chosen as a cutoff; e-values were successively lowered until a sequence or sequences had sufficient similarity to pass the filter. The candidate PROPEP(s) was selected if it had the highest e-value. For this reason some PROPEPs may have been missed in the queried genomes. For each PROPEP sequence, the C-terminal residues, specifically the functional polypeptide sequences that are likely to act as Pep signaling molecules, were aligned with previously published solanaceous Peps from *S. lycopersicum*,* S. melongena*,* S. tuberosum*, and *Nicotiana spp.* [42], to identify Pep sequences from these progenitor proteins. We also queried an annotated proteome and an annotated genome for *Coffea canephora* (commercially cultivated coffee) and a draft genome for *Coffea humblotiana* (a rare wild coffee species) [17, 43]. Furthermore, NCBI has sequences from *S. pennelli*,* S. commersonii*,* Capsicum baccatum*, and *N. otophora* in its sequence databases; candidate PROPEPs were identified for these species as well.

### Analysis of pep sequences

Alignments and phylogenetic analyses: MSA of PROPEP sequences was carried out by MEGA X [40] with MUSCLE (gap opening penalty − 2.9, gap extension penalty 0, hydrophobicity multiplier 1.2, clustering method was UPGMA). Pairwise alignment of coffee PROPEPs was carried out as above [40]; MSA of *Nicotiana* PROPEPs was carried out as above with the same parameters; Peps MSAs were generated as above with the same parameters. Each solanaceous PROPEP sequence and its source, and each Pep sequence used in the present study, are listed in Supplementary Table 1. The entire list of PROPEP sequences included in the present work, including sequences from *Brassicaceae* are listed as a FASTA file in Supplementary File 4. All *Brassicaceae* sequences were obtained from Lori et al. [10] and are named according to that work. The alignment used in the construction of the phylogram in Fig. 8 is provided in FASTA format in Supplementary File 5. Figure 5 graphic was generated by ExPASy (https://web.expasy.org/translate/) [45].

Sequence logo was generated by the Skylign webserver (http://skylign.org) [13] using MSA shown in Fig. 1A. The profile HMM with default HMMER parameters was the option used to specify stack height in the logo. Because of the lack of significant gaps, the occupancy, insert probability, and expected insert length heat map rows were not included in the graphic.

Metapredict [44] was used to evaluate whether SlPep was also disordered in comparison to systemin, which has previously shown to be a disordered polypeptide. Consensus prediction of order/disorder in SlPep and systemin sequences was performed on the Metapredict webserver [44]. Structure prediction of SlPep was carried out by the PEP-FOLD webserver [23] using the “long” simulation analysis option. The highest scoring 5 models (results) are provided as a PDB file in Supplementary File 1.

### Analysis of phylogeny of PEPRs and related proteins

MSAs of LRR-RLKs from tomato and *Arabidopsis* were generated as above and additionally using ClustalW as the algorithm for alignment. Supplementary Fig. 1 is based on an MSA generated with MEGA X’s default ClustalW parameters. MSA used to generate Fig. 7 is available in the Supplementary Materials (Supplementary File 2). Sequences of all SlGCs and tomato and *Arabidopsis* LRR-RLKs used to generate this MSA are listed in Supplementary File 3.

### Electronic supplementary material

Below is the link to the electronic supplementary material.

Supplementary Material 1

Supplementary Material 2

Supplementary Material 3

Supplementary Material 4

Supplementary Material 5

Supplementary Material 6

Supplementary Material 7

Supplementary Material 8

Supplementary Material 9

## Data Availability

Availability of data and materials: The datasets generated and/or analysed during the current study are available from the corresponding author upon reasonable request. Interactive phylogenetic trees for analyzed LRR-RLKs/GCs and PROPEPs are available at https://itol.embl.de/shared/CjjCRJ1zZHdL hosted by the Interactive Tree of Life.
