# Supplementary material for: Pathogen elicitor peptide (pep), systemin, and their receptors in tomato: sequence analysis sheds light on standing disagreements about biotic stress signaling components
Source: BMC Plant Biol. 2024 Jul 30;24:728. doi: 10.1186/s12870-024-05403-y (PMC11289955; doi:10.1186/s12870-024-05403-y)

Supplementary Figure 2. Full tree of selected LRR-RLKs and tomato GCs: relationship between SlPEPR, SlGC17, and several selected LRR-RLKs and GCs. Stringent parameters for MSA construction (gap opening penalty -2.9, gap extension penalty 0, hydrophobicity multiplier 1.2) using ClustalW algorithm. The evolutionary history was inferred by using the Maximum Likelihood method and JTT matrix-based model. At each branch, the tree with the highest log likelihood is shown. Underneath each branch the fraction of trees in which the associated taxa are clustered together is shown (based on 500 bootstraps). This analysis used 112 amino acid sequences. There were a total of 1204 positions in the final dataset.


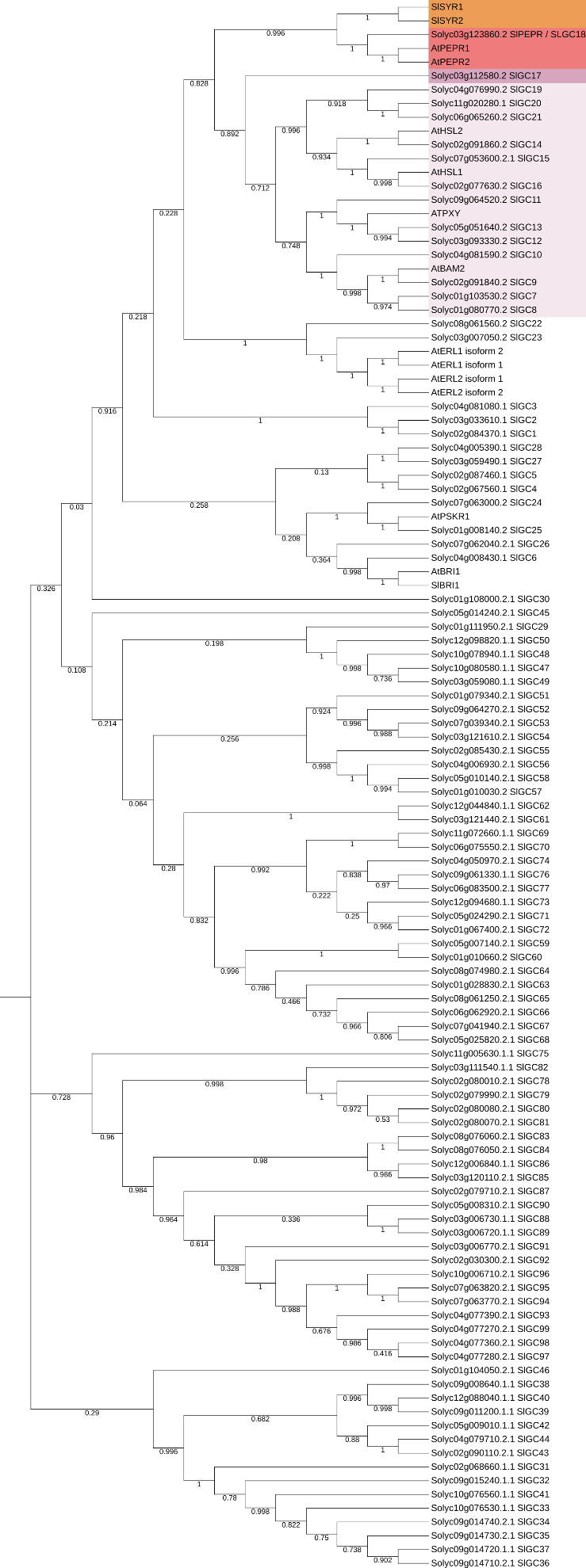

Supplement: Supplementary file 1 — Supplementary Material 1 [file 12870_2024_5403_MOESM1_ESM.docx]
