# Supplementary material for: Pathogen elicitor peptide (pep), systemin, and their receptors in tomato: sequence analysis sheds light on standing disagreements about biotic stress signaling components
Source: BMC Plant Biol. 2024 Jul 30;24:728. doi: 10.1186/s12870-024-05403-y (PMC11289955; doi:10.1186/s12870-024-05403-y)
Supplement: Supplementary file 2 — Supplementary Material 2 [file 12870_2024_5403_MOESM2_ESM.docx]

Supplementary Figure 3


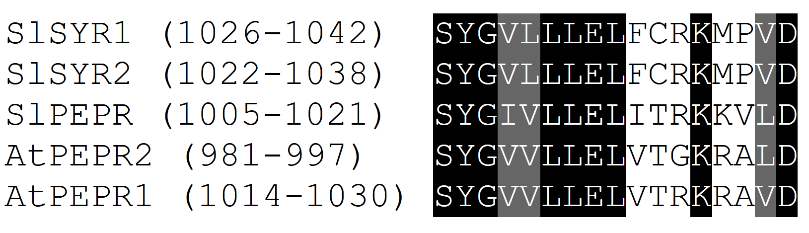
MSA of putative GC center of systemin receptors SlSYR1 and SlSYR2, SlPEPR, and *Arabidopsis* PEPRs AtPEPR1 and AtPEPR2. This MSA provides evidence that residues corresponding with the GC center in SYR and PEPR sequences are conserved. The positions of the residues depicted are shown in parentheses after each sequence name. Identical (matching) residues at each position are highlighted in black, and residues which are chemically similar are highlighted in gray (V/I/L). This MSA is a subset of the MSA supplied in Supplementary File 2 with gaps removed at all-gap positions.
