# Supplementary material for: Pathogen elicitor peptide (pep), systemin, and their receptors in tomato: sequence analysis sheds light on standing disagreements about biotic stress signaling components
Source: BMC Plant Biol. 2024 Jul 30;24:728. doi: 10.1186/s12870-024-05403-y (PMC11289955; doi:10.1186/s12870-024-05403-y)
Supplement: Supplementary file 6 — Supplementary Material 6 [file 12870_2024_5403_MOESM6_ESM.rtf]

Supplementary Figure 1.

Multiple sequence alignments of Pep sequences. Residues which are identical in 7 of the 9 solanaceous sequences are highlighted in black at those positions in the Pep sequences. At these positions, residues with similar chemical properties are highlighted in gray (R/K and I/V). The consensus line at the bottom shows “*” for each position in the alignment that is identical in every sequence, and the “^” symbol indicates positions at which chemical properties of residues are similar among solanaceous sequences and the Arabidopsis sequences. A. MSA of solanaceous Peps and AtPep1-8. The MSA has a total of 25 positions. B. MSA of solanaceous Peps, Coffea Peps, and AtPep1-8.

A. MSA of solanaceous and Arabidopsis Peps.
Capsicum annuum    ATGRRRGR-PPSRPGVGRGPPPENN
C. baccata    	 ATSRRRGR-PPSRPGVGRGPPPENN
C. chinensis    	 ATGRRRGR-SPSRPGVGRGPPPENN
Nicotiana          AT--RRGRTPP-RPGVSRGSPPQNN
Petunia            ATG-RRGRTPP-KSGISQGSPPQHN
Potato group       ATE-RRGR-PPSRPKVGSGPPPQNN
S. chilense Pep b  ATD-RRGR-PPSRPKVVSGPPPQNN
S. melongena	 ATR-RRGR-PPPKPPIGSGSPPQNN
Tomato group       ATD-RRGR-PPSRPKVGSGPPPQNN	 ATR-RRGR-PPPKPP-IGS-GSPPQ-N---N
AtPep1		 ATK-VKAK-QRGKEKVSSGRPGQHN
AtPep2		 DNK-AKSK-KRDKEKPSSGRPGQTN
AtPep3		 EIK-ARGK-NKTKPTPSSGKGGKHN
AtPep4		 GLP-GKKNVLK-KSRESSGKPGGTN
AtPep5		 SLN-VMRKGIR-KQPVSSGKRGGVN
AtPep6		 ITA-VLRRRPR-PPPYSSGRPGQNN
AtPep7		 VSGNVAARKGK-QQT-SSGKGGGTN
AtPep8		 GVI-VKSKKAA-RELPSSGKPGRRN

consensus                 ^          * ^   *


B. MSA of solanaceous, Coffea, and Arabidopsis Peps. 

Coffea canephora    	 A-G--R-GRTPP-RPG-I-RTGN-P-QT-NSSSS
Capsicum annuum    	 ATGR-RRGR-PPSRPG-VGR-GPPP-E-NN----
Capsicum baccata   	 ATSR-RRGR-PPSRPG-VGR-GPPP-E-NN----
Capsicum chinensis	 ATGR-RRGR-SPSRPG-VGR-GPPP-E-NN----
Nicotiana         	 AT---RRGRTPP-RPG-VSR-GSPP-Q-NN----
Petunia            	 ATG--RRGRTPP-KSG-ISQ-GSPP-Q-HN----
Potato group             ATE--RRGR-PPSRPK-VGS-GPPP-Q-NN----
S. chilense Pep b  	 ATD--RRGR-PPSRPKVVS--GPPP-Q-NN----
S. melongena         	 ATR--RRGR-PPPKPP-IGS-GSPP-Q-NN----
Tomato group       	 ATD--RRGR-PPSRPK-VGS-GPPP-Q-NN---- ATR-RRGR-PPPKPP-IG
AtPep1		 	 ATK-VK-AK-QRGKEK-VSS-GR-PGQH-N----
AtPep2		 	 DNK-AK-SK-KRDKEK-PSS-GR-PGQT-N----
AtPep3		 	 EIK-AR-GK-NKTKPT-PSS-GK GGKH-N----
AtPep4		   	 GLP-GKKNVLKKSRE---SS-GKPGGT--N----
AtPep5		       SLN-VMR-KGIR-KQPV-SS-GKRGGV--N----
AtPep6		 	 ITA-VLR-RRPR-PPPY-SS-GRPG-Q-NN----
AtPep7		 	 VSGNVAA-RKGK-QQT--SS-GKGGGT--N----
AtPep8		 	 GVI-VKS-KKAA-RELP-SS-GKPGRR--N---

consensus                        ^            * ^     *

					    
